# Neural crosstalk between subchondral bone and synovium in osteoarthritis

**DOI:** 10.3389/fmed.2026.1971392

**Published:** 2026-10-21

**Authors:** Yiran Feng, Qi Gao, Xiaoya Liu, Yapeng Li

**Affiliations:** 1 Western Sydney University, Sydney, NSW, Australia; 2 Evidence-based Medicine Center, Luoyang Orthopedic Hospital of Henan Province (Orthopedic Hospital of Henan Province), Luoyang, Henan, China; 3 School of Nursing, Fujian University of Traditional Chinese Medicine, Fuzhou, Fujian, China

**Keywords:** neuroimmune crosstalk, neurovascular remodeling, osteoarthritis, sensory nerves, subchondral bone, sympathetic nerves, synovium

## Abstract

Osteoarthritis is a whole-joint disease involving articular cartilage, subchondral bone, synovium, vasculature, and peripheral nerves. However, whether joint innervation merely conveys nociceptive input or actively contributes to disease progression remains unresolved. This review integrates evidence on subchondral bone remodeling, synovial inflammation, neurovascular reorganization, and pain sensitization, and proposes a bidirectional neural crosstalk model between subchondral bone and synovium. In subchondral bone, abnormal mechanical loading and osteoclast activation promote sensory nerve ingrowth and neurovascular remodeling through Netrin-1–deleted in colorectal cancer receptor signaling, prostaglandin E2–prostaglandin E receptor 4 signaling, and Slit guidance ligand 3-related pathways. In the synovium, nerve growth factor and inflammatory mediators reinforce sensory sprouting, neuronal excitability, and neuropeptide release. Calcitonin gene-related peptide, substance P, norepinephrine, and neuropeptide Y act on osteoclasts, osteoblast-lineage cells, fibroblast-like synoviocytes, macrophages, and endothelial cells, thereby linking neural activity to bone turnover, synovial immunity, and vascular remodeling. These signals converge on the receptor activator of nuclear factor-κB ligand–osteoprotegerin axis, nuclear factor-κB, and vascular endothelial growth factor-related pathways. Osteochondral channels, periosteal–synovial interfaces, and neurovascular networks may provide anatomical routes for this cross-compartment communication, forming a feedback circuit that sustains structural damage and chronic pain. Neural effects vary across disease stages and tissue compartments, and pain relief does not necessarily confer structural protection. Future therapies should therefore prioritize stage-specific and compartment-selective neuromodulation while assessing pain, function, and joint structural safety in parallel.

## 1 Introduction

Osteoarthritis (OA) was traditionally viewed as a disorder of cartilage degeneration. It is now recognized as a whole-joint disease involving cartilage, subchondral bone, synovium, the vasculature, and nerves (1, 2). Multi-tissue transcriptomic profiling has identified molecular subtypes characterized by metabolic, inflammatory, and sensory-neural signatures and has revealed ligand-receptor communication among cartilage, subchondral bone, and synovium (3). Single-cell and spatial transcriptomic studies further show that synovial cells can influence chondrocyte states; OA joints contain fewer reparative stromal cells, more proinflammatory and pain-associated macrophages, and spatially localized immune infiltrates (4–6). Together, these findings support a view of OA as a disease driven by disrupted homeostasis across multiple tissues and impaired signaling between joint compartments.

Joint innervation provides an anatomical basis linking tissue damage, immune responses, and pain perception. In mouse models of OA, nociceptors sprout into inflamed synovium early in disease; nerve-containing channels in subchondral bone increase as structural damage progresses, and nerve ingrowth is also evident in osteophytes (7). In human knee OA, synovial expression of calcitonin gene-related peptide (CGRP) is associated with pain severity, whereas tyrosine hydroxylase (TH) and β2-adrenergic receptor (β2-AR) expression are increased in load-bearing subchondral bone (8, 9). A macrophage-nociceptor unit has also been identified around synovial capillaries (10). At the osteochondral interface, extracellular ribonucleic acid (RNA)-vascular endothelial growth factor (VEGF) complexes promote pathological neurovascularization; VEGF receptor 1 (VEGFR1) and VEGF receptor 2 (VEGFR2) signaling preferentially regulate sensory nerve ingrowth and cartilage damage, respectively, whereas simultaneous inhibition of neural and vascular growth reduces hyperalgesia and partly limits structural pathology (11–13). In addition, suppressing proinflammatory polarization of synovial macrophages reduces Netrin-1 expression and CGRP-positive nerve ingrowth, while extracellular vesicle-mediated delivery of microRNAs attenuates nerve growth factor (NGF)-induced sensory-neuron sensitization (14, 15). These findings suggest that the articular neurovascular network not only conveys injury-related signals but also contributes actively to persistent inflammation, tissue remodeling, and chronic pain.

However, studies have largely examined subchondral neurovascularization, synovial neuroimmune responses, sympathetic regulation, and pain sensitization in isolation. The sequence and relative contribution of signals exchanged between compartments therefore remain unclear. A causal role for dorsal root ganglion (DRG) macrophages in maintaining OA pain is supported by cell-depletion experiments, which also show that pain relief can occur without parallel improvement in structural damage (16). Osteoclast-derived Netrin-1 has likewise been shown, genetically and pharmacologically, to promote sensory nerve ingrowth through the deleted in colorectal cancer receptor (DCC) (17). By contrast, the causal contribution of osteochondral channels, the role of Slit guidance ligand 3 (SLIT3)/Roundabout homolog 1 (Robo1) signaling in sensory axon guidance in knee OA, and the limits of NGF-induced structural remodeling remain unresolved (18–20). The available evidence therefore does not support classifying any neural pathway as uniformly pathogenic or protective; its effects are likely to depend on the tissue compartment, disease stage, and local signaling environment.

This review examines bidirectional neural crosstalk between subchondral bone and synovium in OA, whereby tissue-derived signals modify joint innervation and neuronal activity, while neural mediators reciprocally regulate bone remodeling and synovial responses. Within this framework, we discuss neural remodeling, neuropeptide and neurotransmitter signaling, and the receptor activator of nuclear factor-κB (RANK)/receptor activator of nuclear factor-κB ligand (RANKL)/osteoprotegerin (OPG) axis and nuclear factor-κB (NF-κB) as convergent signaling nodes. We then assess neural-targeted strategies for pain relief, functional improvement, and structural safety, together with the main barriers to clinical translation.

## 2 Pathophysiological features of subchondral bone and synovium in OA

### 2.1 Aberrant remodeling and nerve ingrowth in subchondral bone

Subchondral bone remodeling in OA is characterized by increased osteoclast activity and bone resorption early in disease, followed by trabecular thickening and sclerosis; bone marrow lesions and osteophytes are also common features (21, 22). Abnormal mechanical loading promotes osteogenic and angiogenic responses involving transforming growth factor-β (TGF-β) and VEGF (23). Under physiological conditions, sensory and sympathetic nerves innervate subchondral bone, synovium, and the joint capsule, whereas non-calcified articular cartilage is largely aneural (24, 25) (Figure 1A). Neural remodeling in OA involves changes in both sensory and sympathetic innervation, with regional interactions also reported in temporomandibular joint OA (26).

**Figure 1:**
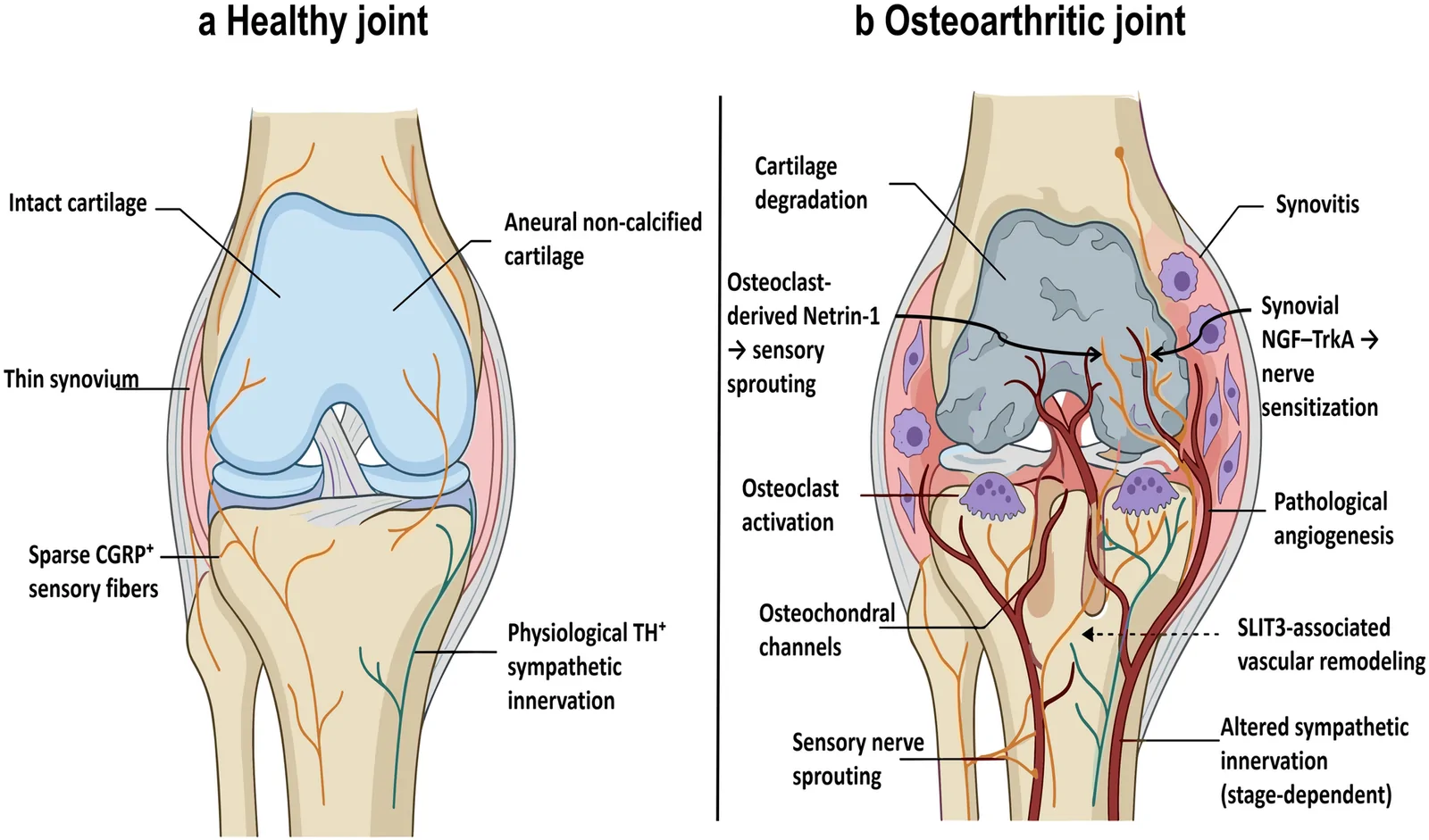
Neural and neurovascular remodeling in healthy and osteoarthritic joints. **(a)** In the healthy joint, the articular cartilage remains intact and the non-calcified cartilage is aneural. The synovium is thin, CGRP-positive sensory fibers are sparse, and TH-positive sympathetic innervation is maintained within a physiological distribution. **(b)** OA is characterized by cartilage degradation, synovitis, osteoclast activation, pathological angiogenesis, and the formation of osteochondral channels. Osteoclast-derived Netrin-1 promotes sensory nerve sprouting, whereas synovial NGF activates TrkA signaling and enhances sensory nerve sensitization. SLIT3-associated vascular remodeling may facilitate neurovascular reorganization in subchondral bone. Sensory fibers extend toward the osteochondral interface and inflamed synovium, while sympathetic innervation undergoes stage-dependent changes in density and spatial distribution. Together, these alterations link subchondral bone remodeling, synovial inflammation, and nociceptive signaling in OA. CGRP, calcitonin gene-related peptide; NGF, nerve growth factor; OA, osteoarthritis; SLIT3, Slit guidance ligand 3; TH, tyrosine hydroxylase; TrkA, tropomyosin receptor kinase A Created with BioRender.com.

Osteoclast-derived Netrin-1 promotes sensory nerve ingrowth through DCC (17), while SLIT3 contributes to vascular remodeling in knee OA and sensory nerve growth in temporomandibular joint OA models (18, 27). Prostaglandin E2 (PGE2) activates prostaglandin E receptor 4 (EP4) in osteoclasts, promoting platelet-derived growth factor-BB (PDGF-BB) release, H-type vessel formation, and Netrin-1-associated sensory innervation (28). Osteoblasts also participate directly in neural signaling. In experimental OA, osteoblast-derived PGE2 acts on neuronal EP4, and osteoblast-specific deletion of cyclooxygenase-2 (COX-2) reduces OA-related pain (29). PGE2 therefore influences sensory nerves through both osteoclast-mediated remodeling and direct neuronal signaling.

Osteocytes regulate osteoclastogenesis through RANKL (30) and may thereby influence osteoclast-dependent nerve ingrowth. They also produce sclerostin, encoded by the sclerostin gene, which inhibits Wnt signaling and bone formation. In human knee OA specimens, lower osteocytic sclerostin expression is associated with more severe cartilage damage and increased subchondral bone mass (31). These observations suggest that osteocytes may influence neural remodeling indirectly through changes in bone turnover, although direct effects of osteocyte-derived RANKL or sclerostin on joint innervation in OA have yet to be established.

Neural signaling, in turn, regulates joint tissue responses. Sympathectomy aggravates subchondral sclerosis in mice subjected to destabilization of the medial meniscus (DMM) (32). In aging mice, norepinephrine (NE) activates chondrocyte β1-adrenergic signaling and promotes exosomal microRNA-125 transfer, disrupting subchondral bone homeostasis (33). Sensory denervation can also worsen cartilage degeneration and synovitis in early experimental OA (34). These findings indicate that neural activity can both support joint homeostasis and contribute to pathological remodeling, depending on the experimental model, disease stage, and tissue compartment.

### 2.2 Synovial inflammation: chronic low-grade inflammation drives joint damage and pain

The synovial lining is among the first joint tissues to respond in OA. In healthy joints, it comprises one to two layers of macrophages and fibroblasts, while the sublining contains blood vessels, lymphatic vessels, and a small number of unmyelinated C fibers (35). OA synovium is characterized by multilayered lining hyperplasia, infiltration by macrophages and T lymphocytes, neovascularization, and fibrosis (36). These changes can be detected early, before marked cartilage degeneration becomes evident (37).

Activated fibroblast-like synoviocytes (FLS) and macrophages release interleukin-1β (IL-1β), tumor necrosis factor-α (TNF-α), and interleukin-6 (IL-6), together with matrix metalloproteinases (MMPs) that directly degrade the extracellular matrix of articular cartilage (38). Damage-associated molecular patterns (DAMPs) released from degenerated cartilage and extracellular matrix activate pattern-recognition receptors on synovial macrophages and amplify inflammatory signaling (39). In a longitudinal study of radiographic knee OA progression in individuals with previous meniscectomy, higher baseline synovial fluid concentrations of IL-6 and TNF-α were associated with subsequent osteophyte progression (40). This observational association does not establish a direct causal role for TNF-α in osteophyte formation. Continued matrix degradation generates further damage signals, stimulating additional release of inflammatory mediators and proteases and allowing molecular injury to precede radiographic change (41).

Synovial inflammation is also an independent source of OA pain. IL-1β and TNF-α can directly lower the activation threshold of articular nociceptors, while binding of C-C motif chemokine ligand 2 (CCL2) to C-C motif chemokine receptor 2 increases sensory-neuron excitability (35). In experimental synovitis, endogenous adenosine triphosphate activates purinergic P2X receptor 3 and heteromeric purinergic P2X2/3 receptors, promoting inflammatory mediator release and neutrophil migration and thereby indirectly sensitizing primary afferent fibers (42). Changes in synovial nerve fibers are disease-stage- and model-dependent: focal nerve growth can occur in actively inflamed regions and correlate with pain, whereas reduced fiber density and disorganized innervation have also been observed in spontaneous or late-stage disease (43–45). Thus, abnormal innervation, rather than a uniform increase in nerve density, is more likely to contribute to reciprocal amplification of synovitis and pain.

Together, aberrant nerve ingrowth in subchondral bone and chronic synovial inflammation form two central components of OA tissue remodeling (Table 1). The osteochondral interface, the periosteal-synovial junction at the joint margin, and mediator transport through synovial fluid connect these pathological processes. OA remodeling therefore extends beyond cartilage loss to include abnormal subchondral bone turnover, synovial inflammation, pathological angiogenesis, and disordered innervation (Figure 1B).

**Table 1:** Pathological features of neural remodeling in subchondral bone and synovium in OA.

Dimension	Subchondral bone	Synovium	Ref
Core pathology	Imbalanced bone turnover: increased bone resorption at an early stage, followed by trabecular thickening and sclerosis.	Chronic low-grade inflammation: lining hyperplasia, immune-cell infiltration, and fibrosis.	(17, 46, 47)
Key cells	Osteoclasts, osteoblast-lineage cells, osteocytes, and endothelial cells.	FLS, synovial macrophages, T cells, and mast cells.	(17, 46, 47)
Key signals	RANKL, TGF-β, PGE2/EP4, and VEGF-related vascular signaling.	IL-1β, TNF-α, IL-6, DAMPs, and NGF.	(28, 45, 47)
Neural remodeling	Osteoclast-derived Netrin-1 promotes sensory axon sprouting through DCC; SLIT3 primarily contributes to H-type vessel formation and neurovascular remodeling.	NGF promotes sensory nerve sprouting and sensitization; NTN4 and Netrin-related signals suggest that synovial stromal and endothelial cells may contribute to nerve growth.	(18, 48, 49)
Changes in innervation	CGRP-positive fibers increase with active bone resorption, angiogenesis, and formation of osteochondral channels.	The density and spatial distribution of sensory and sympathetic nerves change in a disease-stage- and model-dependent manner.	(17, 50, 51)

CGRP, calcitonin gene-related peptide; DAMPs, damage-associated molecular patterns; DCC, deleted in colorectal cancer receptor; EP4, prostaglandin E receptor 4; FLS, fibroblast-like synoviocytes; IL-1β, interleukin-1 beta; IL-6, interleukin-6; NGF, nerve growth factor; NTN4, netrin-4; OA, osteoarthritis; PGE2, prostaglandin E2; RANKL, receptor activator of nuclear factor-κB ligand; SLIT3, slit guidance ligand 3; TGF-β, transforming growth factor beta; TNF-α, tumor necrosis factor alpha; VEGF, vascular endothelial growth factor.

## 3 Neuroimmune remodeling of the synovium

### 3.1 Pathological sensory nerve growth drives joint pain sensitization

Several factors released within synovial lesions can drive pathological sensory nerve growth and contribute to pain sensitization in OA. In hypertrophic synovium from patients with hip OA, the density of sensory fibers positive for protein gene product 9.5 is greater than in non-hypertrophic synovium and is associated with pain severity (52). In rats with medial meniscectomy-induced OA, synovial NGF increased early, followed by a peak in CGRP-positive nerve density in the suprapatellar region at week 4 and a decline by week 6, whereas sensory innervation in osteochondral channels and bone marrow continued to increase (53). Binding of NGF to tropomyosin receptor kinase A (TrkA) upregulates transient receptor potential vanilloid 1 (TRPV1) and promotes nerve ingrowth; TrkA blockade suppresses both nerve growth and hyperalgesia (54). In inflammatory arthritis models, anti-NGF treatment also reduces sprouting of synovial CGRP-positive, neurofilament 200-positive, and sympathetic fibers and relieves pain (55), although these findings cannot be directly extrapolated to structural OA.

FLS also contribute to sensory nerve remodeling. In synovial tissue from patients with end-stage knee OA, Netrin-4 (NTN4) expression correlates with pain scores. Recombinant NTN4 promotes neurite extension in sensory neurons derived from human induced pluripotent stem cells and stimulates OA FLS to release IL-6 and interleukin-8 (56). Activated endothelial cells in fibrotic synovium release Netrin-1, which induces nerve sprouting through DCC receptors on dorsal root ganglion neurons. This response is accompanied by increased peripheral CGRP, substance P (SP), and NGF and greater pain sensitivity (57).

### 3.2 Sympathetic regulation of synovial inflammation in OA

Local sympathetic signaling can modulate synovial inflammation in OA. In rats with monoiodoacetate-induced knee OA, electroacupuncture increased synovial NE levels and reduced macrophage infiltration and proinflammatory cytokine production. Local sympathetic blockade or antagonism of β2-adrenergic receptors (β2-ARs) attenuated these effects, indicating that noradrenergic signaling contributed to the suppression of synovitis (58). In a mouse model of post-traumatic OA, intra-articular administration of the β-adrenergic agonist isoproterenol suppressed inflammatory and profibrotic gene expression in synovium and infrapatellar fat pad tissue from injured female mice. Comparable responses were not observed in males, suggesting that sex influences the local response to adrenergic stimulation (59). However, chemical sympathectomy in male mice subjected to destabilization of the medial meniscus eliminated synovial sympathetic fibers without significantly changing synovitis severity (32). These studies implicate sympathetic signaling in the regulation of synovial inflammation and fibrosis-related gene expression, with outcomes varying according to sex, the OA model, and the intervention used.

### 3.3 Neurovascular amplification of synovial neuroimmune signaling

Synovial neural remodeling is closely associated with angiogenesis. VEGF and NGF are concomitantly upregulated in OA synovium, and newly formed vessels provide a physical scaffold for sensory-fiber extension. CGRP+ fibers, normally restricted to the synovial sublining, may grow along blood vessels into the lining layer and synovial villi (60). This coordinated neurovascular remodeling converts local angiogenic signals into persistent structural abnormalities of innervation. Expanded sensory terminals release CGRP and SP, which act on their corresponding receptors on FLS and macrophages. In turn, activated stromal and immune cells secrete large amounts of NGF and IL-1β, further inducing nerve sprouting and sensitization and establishing a self-amplifying neuroimmune feedback loop.

## 4 Neurotransmitters and neuropeptides in subchondral bone remodeling

### 4.1 Bidirectional regulation of bone metabolism by CGRP

CGRP is a 37-amino-acid neuropeptide released from sensory nerve terminals and exerts bidirectional effects on bone formation and resorption. Under physiological conditions, CGRP binds to a receptor complex composed of calcitonin receptor-like receptor (CLR) and receptor activity-modifying protein 1 (RAMP1), activating cyclic adenosine monophosphate (cAMP)-protein kinase A (PKA) and mitogen-activated protein kinase (MAPK) signaling. These pathways inhibit osteoclast differentiation and promote osteoblast proliferation and mineralization (61). Mice lacking α-calcitonin gene-related peptide (αCGRP) develop age-related reductions in bone formation, increased osteoclastic activity, and loss of bone mass, supporting a role for CGRP in skeletal homeostasis (62). CGRP also promotes osteogenic differentiation of bone marrow mesenchymal stem cells by phosphorylating cAMP response element-binding protein and upregulating runt-related transcription factor 2 (RUNX2) and osteocalcin (63).

The OA microenvironment alters the biological effects of CGRP. In a monosodium iodoacetate (MIA)-induced rat model of knee OA, CGRP and TrkA are persistently upregulated in DRG neurons innervating subchondral bone, and the magnitude of this increase correlates with the extent of subchondral damage (64). Bone samples from postmenopausal women with OA likewise show associations between the abundance of CGRP-positive nerves, deterioration of bone microarchitecture, and joint pain (65). In a cohort of patients with knee OA, serum and synovial fluid CGRP concentrations were positively associated with radiographic severity, linking higher CGRP levels to greater structural damage (66). Mechanistic studies in age-related OA mice indicate that αCGRP drives aberrant RANKL expression and osteoclastogenesis. Although αCGRP deficiency protects cartilage, it aggravates subchondral sclerosis and trabecular bone loss, providing genetic evidence that CGRP has opposing effects on bone metabolism in OA (67).

The osteogenic effects of CGRP may also contribute to abnormal tissue formation under pathological conditions. CGRP can promote endochondral ossification in periarticular tissues, including ligaments, through VEGF- and focal adhesion kinase-related signaling (68). In senescence-accelerated mice, administration of the CGRP receptor antagonist rimegepant before the development of subchondral sclerosis reduces synovitis, cartilage degeneration, and bone sclerosis, whereas late treatment is less effective (69). The net effect of CGRP is therefore more likely to depend on disease stage, tissue compartment, and signal intensity than to represent a uniformly pathogenic action (Figure 2A).

**Figure 2:**
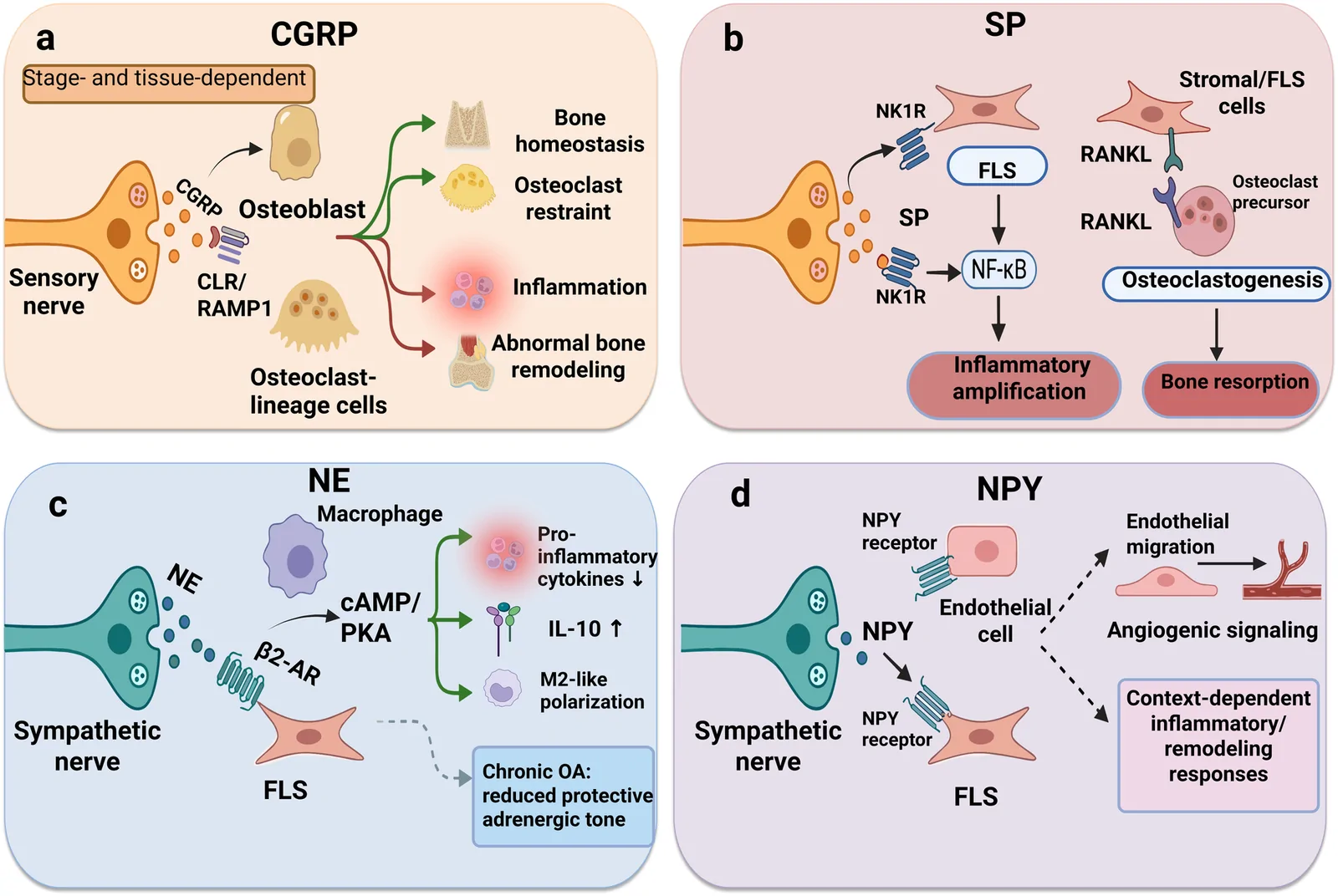
Context-dependent effects of neural mediators in osteoarthritis. **(a)** CGRP regulates bone homeostasis, osteoclast activity, inflammation, and abnormal bone remodeling in a stage- and tissue-dependent manner. **(b)** SP activates NK1R/NF-κB signaling and RANKL/RANK-mediated osteoclastogenesis, promoting inflammatory amplification and bone resorption. **(c)** NE activates β2-AR/cAMP/PKA signaling, suppresses proinflammatory cytokine production, increases IL-10, and promotes M2-like macrophage polarization; this protective adrenergic tone is reduced in chronic OA. **(d)** NPY regulates endothelial migration, angiogenic signaling, and context-dependent inflammatory and remodeling responses in endothelial cells and FLS. β2-AR, β2-adrenergic receptor; cAMP, cyclic adenosine monophosphate; CLR, calcitonin receptor-like receptor; FLS, fibroblast-like synoviocytes; IL-10, interleukin-10; NE, norepinephrine; NF-κB, nuclear factor-κB; NK1R, neurokinin-1 receptor; NPY, neuropeptide Y; PKA, protein kinase A; RAMP1, receptor activity-modifying protein 1; RANK, receptor activator of nuclear factor-κB; RANKL, receptor activator of nuclear factor-κB ligand; SP, substance P. Created with BioRender.com.

### 4.2 Substance P-mediated inflammatory microenvironment in bone

SP is secreted by sensory nerve terminals and acts on subchondral bone through the high-affinity neurokinin-1 receptor (NK1R), creating a local pathological microenvironment characterized by inflammatory activation and excessive bone resorption. NK1R is widely expressed on osteoclast precursors, monocytes, and macrophages. SP binding activates protein kinase C (PKC) and MAPK signaling cascades, promotes the differentiation of bone marrow-derived macrophages into osteoclasts and their migration toward sites of bone resorption, and ultimately facilitates fusion into multinucleated osteoclasts with enhanced resorptive capacity (70). SP also stimulates monocytes and macrophages to secrete TNF-α, IL-1β, and IL-6. These cytokines act on osteoblasts to increase RANKL production, thereby promoting osteoclast differentiation and maturation and aggravating local bone loss (71).

The proinflammatory microenvironment induced by SP can aggravate subchondral bone damage through mutually reinforcing pathways. SP/NK1R signaling activates NF-κB in osteoclast precursors and enhances RANKL-driven osteoclast differentiation (72) (Figure 2B). In parallel, SP stimulates macrophage release of TNF-α and IL-1β, which further sensitize sensory nerves and increase SP release (35). Studies of bone cells from tachykinin precursor 1-deficient mice show that SP deficiency is accompanied by fewer osteoclast precursors, lower cathepsin K activity, and reduced bone resorption, providing additional support for its proresorptive effect (73).

SP/NK1R-related bone inflammation is not specific to OA; in rheumatoid arthritis and periodontitis, it is also associated with NF-κB/RANKL activation and local bone loss (72). In OA subchondral bone, trabecular microfractures and debris generated by cartilage breakdown provide persistent damage signals. Continued SP release from local sensory nerves may therefore sustain and amplify chronic inflammation within the marrow. Small-molecule NK1R antagonists and SP-neutralizing antibodies can reduce inflammation-associated bone loss in animal models (72). In human tissue samples, SP expression was higher in mild OA than in severe OA (74). In ankle OA, subchondral SP expression was associated with bone sclerosis and cartilage degeneration (75). These findings suggest that SP expression varies with tissue location and disease severity.

However, earlier clinical trials of NK1R antagonists in several pain conditions failed to demonstrate analgesic efficacy, highlighting the difficulty of translating preclinical findings into effective pain treatment (50, 76). A recent meta-analysis reported modest reductions in postoperative pain at 2 and 24 h, with no significant reduction in opioid consumption (77). These findings indicate that analgesic effects may vary across clinical settings, and the therapeutic value of NK1R blockade in OA requires further clinical evaluation (78).

## 5 Neurotransmitters and neuropeptides in synovial inflammation

### 5.1 Bidirectional regulation of synovial immune cells by norepinephrine

Sympathetic nerve terminals release NE within the synovium, where it regulates the local immune microenvironment through concentration-dependent receptor selection. At relatively high local concentrations, NE preferentially binds β2-adrenergic receptors on synovial immune cells and activates stimulatory G-protein α-subunit/cAMP/PKA signaling. This pathway suppresses macrophage secretion of the proinflammatory mediators TNF-α and IL-6, induces the anti-inflammatory cytokine interleukin-10, and promotes macrophage polarization toward an alternatively activated phenotype (79) (Figure 2C).

In the chronically inflamed OA microenvironment, FLS and macrophages can upregulate tyrosine hydroxylase and synthesize NE locally as a compensatory response; however, the effect of NE depends on receptor subtype, local concentration, and cellular state (24, 79). In a rat model of knee OA, electroacupuncture enhances local sympathetic noradrenergic signaling and suppresses the C-X-C motif chemokine ligand 1/C-X-C motif chemokine receptor 2 axis through β2-ARs, thereby reducing monocyte/macrophage infiltration and lowering TNF-α and IL-1β levels. Chemical sympathetic blockade or β2-AR antagonism attenuates these effects (58).

These anti-inflammatory effects suggest that reduced local NE availability may weaken adrenergic regulation of synovial inflammation. Evidence from human OA-derived cells supports local catecholaminergic regulation, although its role in patients remains to be established (80, 81). Future studies should examine longitudinal relationships between synovial NE levels, its cellular sources, and synovitis severity, alongside receptor-specific experiments in patient-derived tissue, to determine whether the responses observed *in vitro* also occur *in vivo*.

### 5.2 Regulation of synovial vascular and inflammatory responses by NPY

Neuropeptide Y (NPY) is a 36-amino-acid peptide co-stored with NE in large dense-core vesicles of sympathetic nerve terminals and released during high-frequency activation (82). NPY acts mainly through neuropeptide Y receptor subtypes Y1, Y2, and Y5, with effects that vary by cell type and tissue. Under physiological conditions, NPY is a potent vasoconstrictor that cooperates with NE to maintain basal synovial vascular tone, regulate local blood-flow distribution, and influence immune-cell accumulation within the sublining (79). During OA, sustained sympathetic overactivation may cause excessive local NPY release, shifting its role from vasoconstriction toward pathological angiogenesis (83). Mechanistically, NPY binding to Y1 receptors on FLS can induce VEGF secretion and promote the formation of immature, hyperpermeable vessels, thereby facilitating the entry and retention of monocytes and neutrophils in the synovium and amplifying inflammation (84). However, the complete causal sequence linking sympathetic overactivation directly to synovial angiogenesis in human OA remains insufficiently supported. Therefore, a more appropriate interpretation is that NPY may work in conjunction with NE to regulate synovial blood flow, vascular remodeling, and immune cell recruitment (Figure 2D).

NPY also modulates inflammatory and structural responses in experimental OA. In patients with knee OA, synovial fluid NPY concentrations are elevated and correlate with pain severity and radiographic grade, although these associations do not establish causality (85). In experimental OA, osteocyte-specific Npy deletion attenuated DMM-induced joint pathology, while NPY increased IL-1β, IL-6, and TNF-α expression in RAW264.7 macrophages (86). Conditioned medium from NPY-treated macrophages also promoted osteoclast formation, indicating that some effects may be mediated indirectly through inflammatory signals between joint cell populations (86). Npy silencing reduced inflammatory cytokine expression in FLS derived from mice with collagen-induced arthritis, but this model cannot be directly extrapolated to human OA synovium (87). Thus, causal evidence in human OA remains lacking, and studies using patient-derived synovial tissue are needed to distinguish direct cellular responses from indirect vascular, inflammatory, and bone-mediated effects.

CGRP, SP, NE, and NPY do not have fixed proinflammatory or anti-inflammatory effects. Their actions depend on receptor subtype, target cell, tissue compartment, and disease stage. CGRP and SP are more closely linked to subchondral bone remodeling and amplification of bone inflammation, whereas NE and NPY primarily regulate synovial immunity and vascular responses. Together, these mediators form a tissue-specific and context-dependent neural regulatory network (Figure 2).

## 6 Molecular mechanisms of neural crosstalk between subchondral bone and synovium

### 6.1 Synergistic interactions between neuropeptides and cytokines

Subchondral sensory nerves can release CGRP and SP, which may reach synovial FLS and macrophages through osteochondral channels and the periosteal-synovial interface (88). Mechanical loading-induced PGE2 can enhance sensory input through EP4-dependent bone remodeling and neurovascular coupling (89). In the reverse direction, synovial IL-1β and NGF jointly increase membrane expression of TRPV1 and phosphorylation of the voltage-gated sodium channel NaV1.7 in DRG neurons, lowering their activation threshold (2). Intra-articular injection of NGF alone induces synovial hyperplasia and subchondral bone sclerosis without an external mechanical insult, indicating that once activated, this reciprocal circuit can sustain pathological changes independently (35).

Within the subchondral bone-synovium interface, neuropeptides and proinflammatory cytokines converge at three major signaling nodes. The first is the RANKL/OPG axis. CGRP increases RANKL and suppresses OPG expression, while SP increases RANKL transcription through NK1R/NF-κB signaling (70). IL-1β and TNF-α likewise induce RANKL production by osteoblasts and synovial fibroblasts, and together these signals promote osteoclast activation. The second node is NF-κB. Both SP-driven PKC/MAPK signaling and IL-1β/TNF-α-dependent IκB kinase signaling activate NF-κB, jointly increasing the production of IL-6, TNF-α, and matrix metalloproteinases by synovial cells (90). The third is the proangiogenic VEGF pathway. CGRP and SP increase VEGF, while NPY/Y receptor and IL-6/signal transducer and activator of transcription 3 signaling may also influence angiogenic responses in experimental systems, potentially contributing to abnormal neurovascular growth. Vasoactive intestinal peptide signaling through vasoactive intestinal peptide/pituitary adenylate cyclase-activating polypeptide receptors provides a separate negative regulatory arm by reducing interleukin-12, interleukin-23, and interleukin-17 receptor expression in FLS, although this restraint appears weaker in chronic OA (91).

These molecular interactions may persist after the initiating mechanical stimulus has subsided. DAMPs released from damaged cartilage activate Toll-like receptor 4/NF-κB signaling in synovial macrophages, promoting IL-1β and TNF-α secretion. These cytokines, in turn, sensitize sensory nerves and increase CGRP and SP release, thereby aggravating bone resorption and synovial inflammation and generating additional matrix-derived damage signals (44). This molecular bone-synovium-nerve loop is an important mechanism by which OA progresses from intermittent swelling and pain to sustained structural damage.

### 6.2 “Bidirectional signal-exchange” model at sensory nerve terminals

Sensory innervation of subchondral bone and synovium is not anatomically independent. Histological studies by Aso et al. (92) in patients with knee OA and in rats showed that CGRP+ sensory fibers extend through osteochondral channels from the subchondral marrow cavity toward the joint space. The proportion of channels containing sensory fibers was significantly higher in symptomatic cartilage lesions than in asymptomatic lesions. This channel network may allow mechanical and biochemical signals within bone to be conveyed along axons toward the synovial compartment, providing an anatomical basis for neural crosstalk between subchondral bone and synovium.

Bidirectional molecular signaling underlies this crosstalk. From bone to synovium, abnormal loading-induced PGE2 indirectly promotes sensory nerve ingrowth through EP4-dependent bone remodeling and H-type vessel formation, whereas osteoclast-derived Netrin-1 directly guides axons through neuronal DCC (17, 28, 92). SP released from nerve terminals promotes FLS proliferation and proinflammatory mediator expression through NK1R, and CGRP may further amplify synovial responses through COX-2/PGE2 signaling (93). In the reverse direction, CGRP and SP can directly stimulate synovial macrophages and FLS to release NGF, rather than acting only through cartilage-degradation products (2, 44). NGF binding to TrkA increases TRPV1 and CGRP expression in DRG neurons through retrograde signaling and lowers local pain thresholds (54). Obeidat et al. (19) further showed that repeated intra-articular NGF injection in healthy mice induces synovial hyperplasia, nerve sprouting, thickening of subchondral trabeculae, and osteophyte precursors, but not overt cartilage degeneration. On this basis, subchondral bone-synovium crosstalk can be conceptualized as a bidirectional amplification loop built on osteochondral channels and sensory neural networks, with RANKL/OPG, NF-κB, and VEGF as convergent molecular nodes (Figure 3). However, the strength of evidence differs across the links in this circuit. Netrin-1-DCC and NGF-TrkA are supported by relatively direct genetic or pharmacological interventions, whereas SLIT3-dependent axon guidance, PGE2-mediated axonal growth, and the causal role of osteochondral channels remain model-dependent or largely based on tissue-level associations (Table 2).

**Figure 3:**
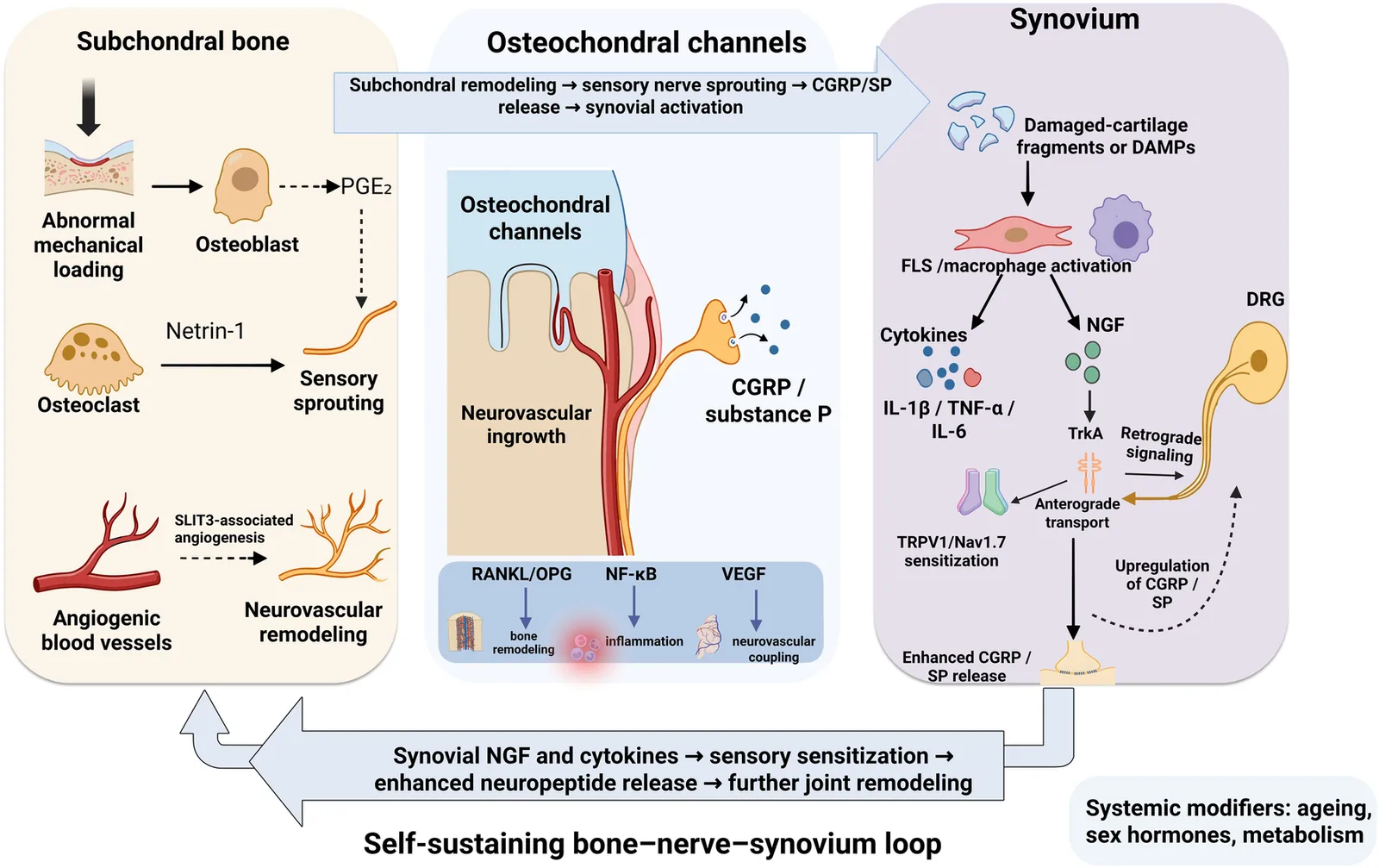
Sensory nerve-mediated crosstalk between subchondral bone and synovium. Subchondral bone: Abnormal mechanical loading promotes bone remodeling, PGE2- and Netrin-1-associated sensory nerve sprouting, and SLIT3-associated neurovascular remodeling. Osteochondral channels: Neurovascular ingrowth through osteochondral channels facilitates CGRP and SP release and is associated with RANKL/OPG, NF-κB, and VEGF signaling. Synovium: Cartilage fragments and DAMPs activate FLS and macrophages, inducing cytokine and NGF production. NGF–TrkA signaling enhances sensory sensitization and CGRP/SP release through communication with the DRG. Overall loop: Synovial inflammation, sensory sensitization, neuropeptide release, and subchondral remodeling may form a self-sustaining bone–nerve–synovium feedback loop. DAMPs, damage-associated molecular patterns; DRG, dorsal root ganglion; IL-1β, interleukin-1 beta; IL-6, interleukin-6; NaV1.7, voltage-gated sodium channel NaV1.7; OPG, osteoprotegerin; PGE2, prostaglandin E2; TNF-α, tumor necrosis factor alpha; TRPV1, transient receptor potential vanilloid 1; VEGF, vascular endothelial growth factor Created with BioRender.com.

**Table 2:** Representative pathways and evidentiary boundaries in neural crosstalk between subchondral bone and synovium.

Key pathway	Principal mechanism	Demonstrated outcomes	Evidence boundaries	Ref
Netrin-1-DCC	Osteoclast-derived Netrin-1 induces CGRP-positive sensory axon ingrowth into regions of active bone resorption.	Netrin-1 deletion or DCC inhibition reduces innervation and alleviates hyperalgesia.	Structural protection is inconsistent; current evidence mainly supports an analgesic effect.	(17)
PGE2-EP4	PGE2 activates EP4 in subchondral osteoclasts, promoting angiogenesis, sensory innervation, and abnormal bone remodeling.	EP4-directed intervention reduces OA pain and improves selected structural measures in animals.	This is an osteoclast-vascular-neural cascade and should not be simplified as direct PGE2-mediated axon guidance.	(28)
SLIT3/Robo1	SLIT3/Robo1 regulates H-type vessels and the neurovascular microenvironment in subchondral bone.	Blockade reduces abnormal vessel formation, bone remodeling, and cartilage degeneration.	Direct evidence for sensory axon guidance in knee OA remains limited.	(18)
Osteochondral channels-CGRP-positive nerves	Nerves and vessels approach the articular surface through osteochondral channels, creating a route for intercompartmental signaling.	The proportion of channels containing CGRP-positive nerves is associated with symptomatic OA lesions in humans and rats.	Current evidence is mainly histological; causal evidence from channel-specific blockade is lacking.	(50)
NGF-TrkA	NGF derived from FLS and synovial macrophages activates TrkA and enhances TRPV1/NaV1.7-related sensitization and DRG signaling.	NGF/TrkA intervention reduces hyperalgesia; sustained NGF exposure induces OA-relevant remodeling.	Complete NGF blockade may impair protective nociception and increase structural joint risk.	(24, 48, 88, 94)

DRG, dorsal root ganglion; NaV1.7, voltage-gated sodium channel NaV1.7; Robo1, roundabout guidance receptor 1; TrkA, tropomyosin receptor kinase A; TRPV1, transient receptor potential vanilloid 1.

These findings support a model in which bone–synovium neural crosstalk includes causal components, although circuit activity remains subject to systemic regulation. Corciulo et al. (95) showed that physiological concentrations of 17β-estradiol preserve subchondral bone mass, reduce synovial hyperplasia and hyperalgesia, and decrease T-cell numbers in inguinal lymph nodes, suggesting that sex hormones can modulate the circuit at a systemic level. Pharmacological studies have shown beneficial effects of CGRP pathway inhibition in experimental OA, whereas a clinical trial of galcanezumab did not demonstrate clear symptomatic benefit. Anti-NGF antibodies improve OA pain in clinical trials but are associated with important joint safety risks (49, 51, 69, 96, 97). Sensory nerves are therefore better viewed as signaling interfaces linking mechanosensation, inflammatory amplification, and tissue remodeling than as isolated pain detectors.

## 7 Neurogenic inflammation drives pain chronification and structural joint damage in OA

### 7.1 Peripheral and central sensitization in the maintenance of chronic pain

OA pain involves peripheral nociceptors and dorsal root ganglia, as well as the spinal cord and supraspinal centers. Persistent nociceptive input from the joint is transmitted to the dorsal horn through C and A*δ* fibers, where SP, CGRP, and CCL2 are released and promote a shift of microglia toward a classically activated proinflammatory phenotype (94). Activated microglia release brain-derived neurotrophic factor (BDNF), which acts through tropomyosin receptor kinase B (TrkB) to downregulate potassium-chloride cotransporter 2 in second-order spinal neurons, thereby weakening *γ*-aminobutyric acid-mediated inhibition. At the same time, N-methyl-D-aspartate receptor-mediated glutamatergic transmission is enhanced, causing wide-dynamic-range neurons to discharge abnormally at high frequency in response to normally innocuous stimuli (45). These processes provide an important neural basis for pain spread, pain at rest, and central sensitization.

### 7.2 Neural signaling and structural joint damage

OA-related pain, reduced activity, and arthrogenic muscle inhibition can jointly reduce periarticular muscle strength, and experimental evidence indicates that muscle weakness itself may promote structural progression. In DMM mice, Kim et al. (98) found that OA-associated quadriceps atrophy was accompanied by activation of an atrogin-1-related ubiquitin degradation pathway and an approximately 21% loss of muscle strength; additional muscle injury further increased Osteoarthritis Research Society International (OARSI) cartilage scores. Conversely, myostatin-deficient mice developed muscle hypertrophy, showed markedly less cartilage degeneration after DMM, and no longer displayed pain-related behavior. From a neuroimmune perspective, mast-cell degranulation mediated by SP and Mas-related G-protein-coupled receptor X2 may amplify synovial inflammation through histamine release and increased vascular permeability, although current evidence comes mainly from mast-cell biology and remains to be validated directly in OA joints (99). Disturbed sympathetic signaling may also contribute to structural damage. Rösch et al. (100) found that β2-AR-deficient mice developed thickening of the calcified cartilage and subchondral bone plate after DMM, together with increased alkaline phosphatase and reduced cathepsin K, suggesting that loss of protective β2-AR signaling shifts subchondral remodeling toward sclerosis.

## 8 Neural modulation-based therapeutic strategies in OA

### 8.1 Pharmacological targeting of neural mediators and receptors

CGRP, SP, and NGF are key mediators linking neurogenic inflammation, peripheral sensitization, and structural changes in the joint. Seidel et al. identified all three as central regulators of chronic pain and neurogenic inflammation, supporting receptor antagonism and ligand-neutralization strategies (101). Clinical studies of NK1R antagonists have yielded mixed analgesic results, and their therapeutic value in OA remains uncertain (50, 76, 77). In an MIA-induced rat model of OA, early intra-articular administration of the NK1R antagonist GR82334 reduced joint inflammation and mechanical hyperalgesia and suppressed neuroimmune responses in the joint, DRG, and spinal dorsal horn, suggesting that this approach may interrupt the transition from local inflammation to persistent pain (102).

Anti-NGF therapy has been evaluated extensively in clinical trials. A meta-analysis of 19 randomized trials found improved Western Ontario and McMaster Universities Osteoarthritis Index pain and function scores, but joint damage increased with dose (97). In pooled phase III data, safety events were dose dependent, and approximately 15% of joints with type 1 rapidly progressive osteoarthritis (RPOA) underwent replacement (96). The mechanism remains uncertain. Complete loss of protective sensation is unlikely because tanezumab does not produce the sensory deficits typical of Charcot arthropathy (47). In unstable animal joints, improved weight bearing during anti-NGF treatment was accompanied by greater cartilage and subchondral bone damage, supporting increased loading after pain relief as a contributing factor (49). NGF blockade could also interfere with cartilage repair and bone adaptation to loading, but direct toxicity to healthy joint tissue has not been demonstrated (47). These studies suggest that RPOA may develop when pain relief increases loading on joints that are already vulnerable to structural damage.

CGRP receptor antagonists offer an opportunity for drug repurposing. Nekomoto et al. (69) showed that the small-molecule antagonist rimegepant reduces OA pain, synovitis, and subchondral sclerosis in mice, with a stronger effect when given early. However, deletion of αCGRP reduces inflammation and cartilage damage while aggravating subchondral sclerosis and bone loss, indicating that CGRP has both proinflammatory and bone-protective functions (67). The anti-CGRP monoclonal antibody galcanezumab also failed to show clear clinical benefit in a randomized trial of knee OA (51). Neural mediator-directed therapy should therefore not rely on complete blockade of a single pathway. Dose, timing, and target tissue must be balanced to relieve pain while preserving joint homeostasis.

### 8.2 Neural-targeted interventions and neuromodulation

Neuromodulation encompasses local drug delivery to regulate ion channels and electrical, thermal, or cryogenic interventions that alter peripheral input and central integration. Chen et al. (103) developed cartilage-targeted, acid-responsive microspheres loaded with carbamazepine, enabling sustained drug release in the acidic OA microenvironment and modulation of NaV1.7-related sodium-channel signaling. In DMM mice, this system increased mechanical pain thresholds, reduced pain-associated neural remodeling and osteophyte formation, and partially preserved joint-space width. However, carbamazepine is not a selective NaV1.7 inhibitor, and its apparent structural effects may also involve regulation of chondrocyte metabolism. For peripheral neural intervention, the increase in CGRP-positive sensory fibers in synovium and subchondral bone provides a pathophysiological rationale for interrupting nociceptive input from the joint. A meta-analysis of eight sham-controlled randomized trials by Barreto et al. (104) showed that genicular nerve radiofrequency ablation and cryoneurolysis reduce pain and improve function for up to 12 weeks, although long-term efficacy and structural benefit remain unclear. For central sensitization, Xia et al. (105) reported that transcutaneous electrical nerve stimulation (TENS) combined with transcranial direct-current stimulation (tDCS) improved pain and walking ability in patients with mild knee OA more than TENS combined with sham stimulation, with benefits persisting for one month after treatment. Transcutaneous vagus nerve stimulation may also enhance descending pain inhibition and activate the cholinergic anti-inflammatory reflex, which suppresses macrophage-derived proinflammatory mediators through α7 nicotinic acetylcholine receptor-related signaling (106). A randomized controlled trial by Elsehrawy et al. (107) showed improvements in visual analog scale pain scores, pressure pain thresholds, central sensitization, neuropathic pain, and Knee injury and Osteoarthritis Outcome Scores, although local anti-inflammatory effects within the joint were not directly assessed.

### 8.3 Blocking pathological nerve growth and multitarget strategies

The strategies described above primarily modulate abnormal neural signals after they have developed, whereas pathological nerve ingrowth may precede persistent sensitization and neurogenic inflammation. Zhu et al. (17) showed that osteoclast-derived Netrin-1 promotes CGRP+ sensory axon ingrowth into OA subchondral bone through DCC. Osteoclast-lineage deletion of Netrin-1 or DCC silencing reduces innervation and alleviates pain without significantly improving cartilage degeneration, indicating that isolated blockade of this axon-guidance pathway is predominantly analgesic. Osteoclast-derived SLIT3 also promotes sensory nerve growth in the osteochondral region of temporomandibular joint OA; specific knockdown or neutralization reduces nerve ingrowth, relieves pain, and attenuates osteochondral damage (27). In a knee OA model, Li et al. demonstrated that inhibition of SLIT3/Robo1 reduces abnormal H-type vessel formation in subchondral bone and improves cartilage degeneration and aberrant bone remodeling (18). These findings suggest that SLIT3 regulates both axon guidance and vascular-bone remodeling, although its effects depend on tissue context and model.

Compared with direct antagonism of individual neuropeptides, limiting pathological neurogenesis may simultaneously reduce the release of CGRP, SP, and other neuropeptides and weaken persistent NGF-TrkA-mediated sensitization, thereby reducing the gain of multiple nociceptive pathways upstream. However, current studies have not established that this strategy fully preserves physiological joint innervation or that multitarget intervention is necessarily superior to monotherapy. Low-dose anti-NGF treatment combined with a CGRP receptor antagonist offers theoretical complementarity by limiting nerve sprouting upstream and suppressing neuropeptide signaling downstream, but OA combination studies in animals and phase I safety data are lacking. This remains a therapeutic hypothesis requiring validation. Current neural-targeted approaches act at different levels, including neural mediators, peripheral sensitization, and neural conduction, but their analgesic benefits, structural effects, and evidentiary maturity are inconsistent (Table 3).

**Table 3:** Representative neural-targeted strategies in OA and their translational limitations.

Representative strategy	Level of action	Current evidence	Principal benefit	Key limitation	Ref
CGRP pathway blockade	Neuropeptide signaling	Positive findings in OA animal studies; an anti-CGRP monoclonal antibody showed no clear benefit in a knee OA trial.	Reduces hyperalgesia, synovitis, and selected subchondral abnormalities in animals.	CGRP contributes to both inflammatory regulation and bone homeostasis; the appropriate patient population and disease-stage window remain undefined.	(51, 69)
Anti-NGF monoclonal antibodies	NGF–TrkA peripheral sensitization	Multiple randomized trials and meta-analyses support improvements in pain and physical function.	Strong clinical analgesic evidence.	Dose-dependent joint safety events and rapidly progressive OA; altered loading of vulnerable joints may contribute, whereas direct toxicity to healthy joint tissue has not been demonstrated.	(49, 96, 97, 108)
SP/NK1R blockade	Neurogenic inflammation	Preclinical studies and clinical analgesic trials across several pain conditions.	Reduces hyperalgesia, synovitis, and neuroimmune activation in animal models.	Clinical analgesic results are mixed, and benefits for OA pain and structural progression remain unproven.	(50, 76, 77, 102)
Genicular nerve ablation	Peripheral nociceptive input	Sham-controlled randomized trials and meta-analyses.	Short-term pain relief and functional improvement.	Long-term efficacy, recurrence, and effects on joint structure remain unclear.	(104)
TENS combined with tDCS	Combined peripheral and central modulation	Small randomized controlled trials.	Improves pain and walking ability in mild-to-moderate knee OA.	Sample sizes are limited, and stimulation parameters and treatment duration are not standardized.	(105)
Transcutaneous vagus nerve stimulation	Autonomic regulation and descending pain inhibition	Preliminary randomized controlled trials.	Improves pain, function, and sensitization-related measures.	Evidence for joint structural effects and local inflammatory biomarkers is lacking.	(107)

NK1R, neurokinin-1 receptor; SP, substance P; tDCS, transcranial direct current stimulation; TENS, transcutaneous electrical nerve stimulation.

## 9 Discussion and future perspectives

Current evidence indicates that neural regulation in osteoarthritis varies with disease stage and tissue context. Early sensory denervation can aggravate cartilage degeneration, whereas αCGRP deficiency reduces inflammation and cartilage damage but may exacerbate subchondral bone sclerosis, suggesting that neural signaling can contribute to disease progression while also supporting joint homeostasis (34, 67). BDNF/TrkB-mediated sensitization changes during disease progression from articular afferents to afferents associated with subchondral bone, and early mechanical unloading can modify inflammatory and structural outcomes (109, 110). Osteochondral channels and sensory neural networks provide a potential anatomical basis for cross-compartment communication between subchondral bone and synovium (92). Abnormal remodeling in subchondral bone can promote sensory nerve ingrowth, whereas synovial NGF and inflammatory signals can in turn enhance neurite sprouting and sensitization, together forming a feedback loop that may sustain structural damage and chronic pain (17, 54). Accordingly, the effects of interventions directed at neural pathways are likely to depend on disease stage, tissue compartment, and the local microenvironment, and analgesic benefit cannot be assumed to confer structural protection (96).

The temporal sequence, cellular sources, and spatial organization of neural remodeling remain incompletely defined. Most animal models capture short disease courses, while human tissue studies are largely cross-sectional (5, 111). A study comparing early and late knee OA found significantly reduced heart rate variability in late OA relative to healthy controls, whereas the reduction in early OA was not significant (112). These systemic measurements provide evidence of autonomic dysfunction but cannot resolve changes in local joint innervation or identify the optimal timing of intervention. Although decay-accelerating factor (CD55)-positive synovial fibroblasts show axon-growth-related features, this evidence derives from rheumatoid arthritis and therefore serves primarily as a methodological reference (113). Coexpression of voltage-gated sodium channel NaV1.8, piezo-type mechanosensitive ion channel component 2, and TrkA has also been demonstrated in human dorsal root ganglion neurons, but information on joint origin and osteoarthritis stage is lacking (114). These limitations make it difficult to distinguish physiological joint innervation from disease-induced pathological neural networks and constrain direct translation of mechanistic findings from animal models to human osteoarthritis.

Future studies should track neural changes within the same patients and compare treatment responses across defined stages of OA. Trials targeting CGRP, SP, or adrenergic signaling should assess both symptom relief and structural outcomes to identify when intervention offers benefit without compromising joint integrity. Integration of single-cell sequencing, spatial transcriptomics, neural tracing, and three-dimensional imaging may clarify how neural ligands, receptors, and target cells change over time and across joint compartments (115, 116). MRI can localize subchondral bone marrow lesions and features of synovitis, but these findings reflect tissue pathology, not neural activity (117, 118). Serum or synovial fluid CGRP measurements cannot distinguish its tissue of origin (66), and quantitative sensory testing does not localize the source of sensitization (119). Combining these assessments with spatial analysis of neural markers in matched bone and synovial samples would help establish their anatomical relevance. Neuropeptide and tissue turnover panels should undergo similar validation before being interpreted as measures of local neural activity (120). Trials of neural stimulation and interventional procedures should include appropriate sham controls and assess pain, function, neural sensitization, MRI-defined structural outcomes, and long-term safety in parallel (121).

## 10 Conclusions

This review highlights evidence for bidirectional, neurally mediated crosstalk between subchondral bone and the synovium, which may represent an important link between structural remodeling and persistent pain in OA. Abnormal subchondral bone remodeling, synovial inflammation, and sensory nerve remodeling may interact through neuropeptides, inflammatory mediators, and neurovascular networks, thereby sustaining cross-tissue signal amplification. Importantly, these neural effects are both disease stage- and tissue compartment-dependent and should not be regarded as uniformly pronociceptive or pathogenic. Joint innervation may therefore function as a regulatory interface linking subchondral bone remodeling, synovial immune responses, and pain sensitization. This concept supports a shift in neural-targeted OA therapy from non-selective analgesia toward stage-specific and tissue-selective modulation.

## Glossary

OA: osteoarthritis
CGRP: calcitonin gene-related peptide
TH: tyrosine hydroxylase
β2-AR: β2-adrenergic receptor
RNA: ribonucleic acid
VEGF: vascular endothelial growth factor
VEGFR1: vascular endothelial growth factor receptor 1
VEGFR2: vascular endothelial growth factor receptor 2
NGF: nerve growth factor
DRG: dorsal root ganglion
DCC: deleted in colorectal cancer receptor
SLIT3: slit guidance ligand 3
Robo1: roundabout homolog 1
RANK: receptor activator of nuclear factor-kappa B
RANKL: receptor activator of nuclear factor-kappa B ligand
OPG: osteoprotegerin
NF-κB: nuclear factor-kappa B
TGF-β: transforming growth factor-β
PGE2: prostaglandin E2
EP4: prostaglandin E receptor 4
PDGF-BB: platelet-derived growth factor-BB
COX-2: cyclooxygenase-2
DMM: destabilization of the medial meniscus
NE: norepinephrine
FLS: fibroblast-like synoviocytes
IL-1β: interleukin-1 beta
TNF-α: tumor necrosis factor-α
IL-6: interleukin-6
MMPs: matrix metalloproteinases
DAMPs: damage-associated molecular patterns
CCL2: C-C motif chemokine ligand 2
TrkA: tropomyosin receptor kinase A
TRPV1: transient receptor potential vanilloid 1
NTN4: netrin-4
cAMP: cyclic adenosine monophosphate
PKA: protein kinase A
MAPK: mitogen-activated protein kinase
αCGRP: α-calcitonin gene-related peptide
MIA: monosodium iodoacetate
SP: substance P
NK1R: neurokinin-1 receptor
PKC: protein kinase C
NPY: neuropeptide Y
Y1/Y2/Y5: neuropeptide Y receptor subtypes 1, 2, and 5
NaV1.7: voltage-gated sodium channel 1.7
BDNF: brain-derived neurotrophic factor
TrkB: tropomyosin receptor kinase B
M1: classically activated microglial phenotype
OARSI: Osteoarthritis Research Society International
RPOA: rapidly progressive osteoarthritis
TENS: transcutaneous electrical nerve stimulation
tDCS: transcranial direct-current stimulation
CD55: decay-accelerating factor
CLR: calcitonin receptor-like receptorx
RAMP1: receptor activity-modifying protein 1.
